# Prolonged Wait Time Prior to Entry to Home Care Packages Increases the Risk of Mortality and Transition to Permanent Residential Aged Care Services: Findings from the Registry of Older South Australians (ROSA)

**DOI:** 10.1007/s12603-018-1145-y

**Published:** 2018-12-04

**Authors:** Renuka Visvanathan, A. T. Amare, S. Wesselingh, R. Hearn, S. McKechnie, J. Mussared, M. C. Inacio

**Affiliations:** 1 National Health and Medical Research Council Centre of Research Excellence in Frailty and Healthy Ageing, University of Adelaide, Adelaide, South Australia Australia; 2 Adelaide Geriatrics Training and Research with Aged Care Centre, School of Medicine, University of Adelaide, Adelaide, South Australia Australia; 3 Aged and Extended Care Services, The Queen Elizabeth Hospital, Central Adelaide Local Health Network, Adelaide, South Australia Australia; 4 Healthy Ageing Research Consortium, Registry of Older South Australians (ROSA), South Australian Health and Medical Research Institute (SAHMRI), Adelaide, South Australia Australia; 5 School of Medicine, University of Adelaide, Adelaide, SA Australia; 6 South Australian Health and Medical Research Institute University (SAHMRI), Adelaide, South Australia Australia; 7 Resthaven Inc., Adelaide, South Australia Australia; 8 Council on the Ageing South Australia (COTA SA), Adelaide, South Australia Australia; 9 Univeristy of South Australia, Division of Health Sciences, Adelaide, South Australia Australia; 10 The Queen Elizabeth Hospital, Level 8B, 28 Woodville Road, Woodville South, Adelaide, SA 5011 Australia

**Keywords:** Healthy ageing, aged care, mortality, nursing home, wait time

## Abstract

**Background:**

Older Australians prefer to live in their own homes for longer and reforms have attempted to increase the volume of home care packages (HCPs) accordingly but there remains a queue with the longer-term consequences unclear.

**Objectives:**

This study aims to characterise older Australians according to their wait times for a home care package (HCP), evaluate the association between wait time and mortality and evaluate the association between wait time and transition to permanent residential aged care services after HCP.

**Design:**

A retrospective cohort study using data from the National Historical cohort (2003-2014) of the Registry of Older South Australians (ROSA) was conducted.

**Setting:**

Home based aged care services, national cohort.

**Methods:**

Wait time was estimated from approval date to date of receiving a HCP. Descriptive, survival estimates (95% confidence intervals (CIs)), and multivariable survival analyses (Cox-regression) were conducted to evaluate the risk of mortality and transition to permanent residential aged care services by quartiles of wait time for HCP.

**Results:**

The cohort was followed for 4.0 years (interquartile range IQR (1.8-7.2)) and 38% were alive at the end of the study period with a median wait time for HCP of 62 (21-187) days. From 178,924 older people who received a HCP during the study period (2003-2013), 33.2% people received HCP within 30 days, 74.3% within 6 months and 25.7% after 6 months. The effect of wait time on risk of mortality was time-dependent, with longer wait times associated with higher mortality in the longer term. Compared to people who waited ≤30 days for a HCP, individuals who waited more than 6 months had an almost 20% excess risk of death (adjusted hazard ratio (aHR), 95%CI = (1.18, 1.16-1.21)) 2 years after entry into a HCP. Those who waited more than 6 months also had a 10% (1.10, 1.06-1.13) higher risk of transition to permanent residential aged care services after two years.

**Conclusion:**

Prolonged wait times for HCP is associated with a higher risk of long-term mortality as well as transition to permanent residential aged care. It remains to be seen if a shortening of this wait time translates into better health outcomes.

**Electronic Supplementary Material:**

Supplementary material is available for this article at 10.1007/s12603-018-1145-y and is accessible for authorized users.

## Introduction

Australia is experiencing population ageing no different to other countries globally. At the end of the 2016/2017 financial year, 15% (3.8 million) of Australia’s population was aged 65 years and older whilst 2% (499,000) was aged 85 years and over (1). By 2017, those aged 85 years and older will make up 2.3% of the population and those aged 65 years and older will form 18% of the population (1). Absolute numbers of older people will correspondingly increase to 672,000 and 5.2 million respectively. Successful implementation of health policy and service models that have kept people healthier for longer is one reason for the population ageing seen globally. It follows that models of care for the future will need to be fit for purpose and meet the heterogeneous health and social care needs of increasing numbers of older consumers whilst balancing challenges arising from competing budgetary pressures, the ageing workforce, urbanization and changing family structures.

It is apparent that older Australians prefer to live in their own homes for longer, closer to family and their community, continue to participate as well as contribute and have a major say in what happens to them (2, 3). In line with their preference, reform to the Australian aged care system has occurred progressively over the last two decades including through a series of 2013 amendments to the Aged Care Act 1997 when the government increased user choice and control of aged care services delivered at home. It has also been previously demonstrated that comprehensive geriatric assessment in the community may contribute to a delay in the development of disability and reduce premature placement into residential aged care services (4). Timely access to appropriate home care packages (HCP) is central to the success of the reform agenda and it may provide older Australians with the best chances at preserving their independence and choice whilst increasing wellbeing (2). This vision is potentially achievable through the national Aged Care Assessment Program (ACAP), which determines eligibility for complex subsidised care as it incorporates a comprehensive health and social assessment by a skilled assessor, which when coupled with a management strategy such as the use HCPs may support older people to live independently for longer (5). HCPs may provide supportive care to some but with others it might also be important in supporting a preventative or restorative approach (6).

In Australia, the supply of subsidised aged care services is determined by the Commonwealth Government by specifying targets and it is, therefore, a capped program. In 2011 for example, for every 1000 people aged 70 years and older, there were 25 home care places and 88 residential aged care places (2). It is anticipated that by 2050, approximately 3.5 million Australians will be tapping into an aged care service each year, with 80% receiving care at home (2). In keeping with the shift to home-based care, the government has taken positive steps with the number of approvals for HCPs, increasing approvals following assessment from 4 out of every 10 in 2013/2014 to 7 out of 10 in 2017/2018 (6). The government aims that by the 2021/2022 financial year, 45 home care places, 78 residential aged care places and 2 short-term restorative care places will be achieved (7). A centralised National Wait List was also developed in February 2017 recording a national picture of unmet need for home care for the first time. Previously individual providers held waitlists and managed prioritisation of progression to an available higher care place based on their knowledge of the needs and risk of their client group. The supply and mix of packages available in each region therefore was different with wait times governed by the complex interaction between supply, mix and demand. As of the 30th June 2018, there were 121,418 consumers on the national prioritisation queue with approximately 47% without access to any form of HCPs with the balance of consumers assigned to lower levels of HCPs or a short-term service through the Community Home Support Programme as an interim strategy (8). The queue for HCPs is a marker of unmet needs in the community. What is unclear is the impact of the waiting time on the future health outcomes of those waiting. Clarification of health outcomes will assist policy makers and clinical providers who are faced with difficult policy and service decisions.

This study aims to look at the historical approvals between July 2003 and June 2013, which would be prior to the implementation of the most recent aged care reforms, and characterise the population by their wait time as well as investigate the effect of the wait times on health outcomes such as mortality and transition to permanent residential aged care services following a period within a HCP.

## Methods

### Definition

The term home care package (HCP) is used in this paper to describe the community-based services funded by the Commonwealth Government. The packages before August 2013 consisted of increasing levels of care from community aged care packages (CACP) through to extended aged care at home (EACH) and extended aged care at home dementia (EACH-D) packages. After August 2013, these packages were replaced by a 4 level system of packages, where Levels 1 and 2 in this paper is equated to the historical CACP and Levels 3 and 4 are equated to the EACH/EACH-D (9).

### Study Setting, Design, and Data Sources

A retrospective cohort study was conducted using data from the National Historical Cohort of the Registry of the Older South Australians (ROSA), which comprised linked data obtained from the Australian Institute of Health and Welfare (AIHW) National Aged Care Data Clearinghouse. Specifically, this study used datasets from the Australian Commonwealth Aged Care Assessment Program (ACAP), HCPs details, residential aged care services details, and National Death Index (NDI) (5, 10, 11). The ACAP de-identified dataset includes every assessment performed by an Aged Care Assessment Team (ACAT) when determining eligibility for aged care services in Australia. These ACAT assessments were undertaken for people seeking permanent or respite residential aged care, HCPs, short-term restorative care or transition care support services. The ACAP dataset contains information on the assessor, service approvals, and aged care seeker. The HCP and residential aged care datasets provide details on the dates (i.e. entry and exits) and levels of services people received. The NDI provide the mortality status for this national cohort.

### Study Population

Our cohort includes people aged 65 years old and older or 50 years old and over for Aboriginal and Torres Strait Islander peoples that had approval for and entered a HCP between July 1st, 2003 and June 30th, 2013 (N=178,924) as the first service received. Individuals who had a wait time of more than 5 years for a HCP and those who may have died whilst waiting or never took up a HCP despite approval were not included.

### Exposure of Interest

Wait time from the ACAT assessment approval to the beginning of a HCP was the main exposure of interest. For people who had several ACAT assessments, we considered the assessment closest to the date of beginning of HCP as the one for this study. Wait time was calculated as the difference in the time between the date of approval and the date of first access to a HCP. The study cohort was divided into four groups (quartiles) according to their wait time and this equated to 0–30 days, 31–59 days, 2–6 months days and >6 months.

### Outcomes of Interest

Mortality after beginning to receive a HCP was the primary outcome of interest. Other outcomes of interest were entry into permanent residential aged care after beginning a HCP and the risk of mortality after transition to permanent residential aged care. Mortality was assessed for the cohort between July 1st, 2003 and June 30th, 2015, yielding a minimum 2 year follow up for the entire cohort, entry into permanent residential aged care was assessed between July 1st, 2003 and June 30th, 2014, yielding a 1 year minimum follow up.

### Covariates

Individuals’ sociodemographic characteristics, living situations, activity limitations, health conditions and geriatric syndrome conditions were evaluated. Specific variables evaluated were: age, sex, English proficiency index/migrants level of English proficiency derived from country of birth (0=Australian born, 1= Countries rating ≥98.5% on the English Proficiency index, 2= Countries rating ≥84.5%, 3= Countries rating 57.5% to less than 84.5%, 4= Countries rating less than 57.5%)(12), country of birth (Australia or born overseas), indigenous status (Aboriginal/Torres Strait Islander or neither), Department of Veterans’ Affairs Card Status (no card, gold card, other), remoteness location (major city vs. other), state, living arrangements (lives alone or with someone), usual accommodation (private owned, retirement village, hospital, short term temporary referring to individuals sheltered in shortterm crisis or emergency or transitional or public places), activity limitations (communication, domestic assistance, health care tasks, home maintenance, meals, movement activities, self-care, social and community participation, transport, moving around places at or away from home), priority category (within 48 hours, between 3 and 14 days, more than 14 days, no priority), other approval (permanent, respite or transition care), HCP entry care level (CACP, EACH or EACH-D), financial year, recommended government assistance services (community aged care, home and community care, veterans’ community aged care service), carer availability, carer co-residency status, carer gender, geriatric syndromes (falls, fractures, delirium, dementia, depression), other health conditions (hypertension, arthritis, diseases of the skin and subcutaneous tissue, cancer, diabetes, diseases of the eye, incontinence, malnutrition, deafness/hearing loss, osteoporosis, chronic lower respiratory diseases, kidney & urinary system disorders, chronic lower respiratory diseases), eligibility assessors’ professional training (medical vs. nursing vs. health vs. social welfare), and current assistance, source of current assistance, and recommended assistance for limitations.

### Analysis

First, the data was cleaned, coded, checked for distributions and then wait time was computed and described by each independent variables. Graphs describe wait time variations by age, gender, financial year and state. To identify factors associated with wait for a HCP, individuals were grouped by quartiles of wait time as 0–30 days vs. 31–59 days vs. 2–6 months vs. > 6 months. Second, a multinomial logistic regression model was fitted using quartiles of wait time as the dependent variable and covariates as a predictor to characterise cohorts according to their wait times. A stepwise variable selection approach was used to determine factors associated with wait time and the best model was determined using the Akaike Information Criterion (AIC). To assess the impact of wait time on mortality, univariate survival analysis was performed followed by a multivariable survival analysis (Cox regression) estimating the risk of mortality in older people who waited for 31–59 days, 2–6 months or > 6 months compared to those who waited 0–30 days. A Cox regression model was also used to examine the effect of wait time on risk of entering residential aged care permanently after a HCP, and evaluate on mortality risk once permanent residential aged care permanently. The findings are described using survival estimates (95% confidence intervals (CI)), Kaplan Meier curve (KM) plots and hazard ratios (HR: 95% CI). Proportional hazard assumptions were evaluated using a scaled Schoenfeld residuals plot and Schoenfeld test, and time-dependent effects were calculated when assumptions were not met (i.e. for the mortality estimates). Cox regression models were adjusted for covariates related to individual’s sociodemographic and personal characteristics known to be associated with wait time (i.e. were known to be associated with wait times). Both adjusted and unadjusted effect estimates (95% CIs) were calculated for estimates of mortality, risk of transition to permanent residential aged care and risk of mortality after entry to permanent residential aged care. Sensitivity analyses were conducted to determine if HCP entry care level (CACP, EACH or EACH-D) was an effect modifier (i.e. interaction) in each of the models. All tests were two-sided and alpha=0.05 was considered statistically significant. The analysis was performed using R programming language version 3.5.1.

## Results

### Characteristics of individuals accessing home care packages

We analysed data from 178,924 older Australians who received a HCP between 2003 and 2013. The mean (SD) age of the cohort was 81.6 (7.0) years. The majority were female (65.2%), born in Australia (66.4%), living in major cities (67.3%), and residing in a private or rental accommodation (87.5%), (Table 1 and Supplementary Table 1). 5855 people entered the newer HCP levels of packages, Level 1 and Level 2 and this was equated to a CACP. 32 people received Level 3 and level 4 packages, and this was equated to EACH/EACH-D.

### Wait times for home care packages

Of the 178,924 older people who received a HCP as their first service during the study period, 33.2% of them received services with 30 days, 41.2% between 30 days to 6 months and 25.6% after 6 months. The overall median wait time was 62 (IQR=21-187) days. Wait time to obtain HCP varied by sex, country of birth, indigenous status, living arrangements, current accommodation, the existence of activity limitations and health conditions, across years and by state (Table 1, Supplementary Table 1, Supplementary Figure 1a-e). Of the 142,755 people who were approved for CACP packages, 137,790 (96.5%) received this package at entry. Among people who were approved for EACH (N=15,712), 4030 (25.6%) received a lower level CACP and of the 7087 approved for EACH-D, 931 (13.1%) received a lower level package initially (Supplementary Figure 1a).

**Table 1:** Characteristics of individuals by quartiles of wait time for home care packages, 2003-2013

**Variables**		**Wait time**			
	**0–30 days**	**31–59 days**	**2–6 months**	**Over 6 months**	
Total cases; N (%) Wait time;	178924 (100)	59366(33.2)	28014(15.7)	45621(25.5)	45923(25.6)
median (IQR) Age	62(21, 187)	13(6,21)	42(36,50)	100(78,133)	358(251,627)
Mean (SD)	81.6 (7.0)	81.7(7.1)	81.8(7.0)	81.6(7.0)	81.3(6.8)

	**Category**	**N (%)**	**N (%)**	**N (%)**	**N (%)**
Sex					
	Female	38156(64.3)	18286(65.3)	30145(66.1)	30151(65.7)
	Male	21125(35.6)	9697(34.6)	15423(33.8)	15735(34.3)
	Missing	85(0.1)	31(0.1)	53(0.1)	37(0.1)
Country of birth					
	Australia	39677(66.8)	18805(67.1)	30311(66.4)	29985(65.3)
	Born overseas	19679(33.1)	9206(32.9)	15303(33.5)	15935(34.7)
	Missing	10(<0.1)	3(<0.1)	7(<0.1)	3(<0.1)
Remoteness					
	Major City	39428(66.4)	19227(68.6)	30968(67.9)	30834(67.1)
	Other	19793(33.3)	8702(31.1)	14503(31.8)	14904(32.5)
	Missing	145(0.2)	85(0.3)	150(0.3)	185(0.4)
Department of veterans’ affairs (DVA) card status					
	DVA gold card	5321(9.0)	2508(9.0)	3977(8.7)	3831(8.3)
	DVA white card	705(1.2)	313(1.1)	580(1.3)	621(1.4)
	No DVA card	49240(82.9)	22988(82.1)	36273(79.5)	36256(78.9)
	Other DVA card	1251(2.1)	516(1.8)	937(2.1)	1136(2.5)
	Missing	2849(4.8)	1689(6.0)	3854(8.4)	4079(8.9)
Entry care level					
	CACP	52602(88.6)	24446(87.3)	38678(84.8)	32334(70.4)
	EACH	3852(6.5)	1979(7.1)	3365(7.4)	4908(10.7)
	EACHD	2756(4.6)	1344(4.8)	1977(4.3)	2679(5.8)
	LEVEL1	0 (0.0)	2 (<0.1)	25(0.1)	148(0.3)
	LEVEL2	23 (<0.1)	156 (0.6)	1195(2.6)	4306(9.4)
	LEVEL3	0 (0.0)	0 (0.0)	1(<0.1)	8(<0.1)
	LEVEL4	0 (0.0)	0 (0.0)	5(<0.1)	18(<0.1)
	Missing	133(0.2)	87(0.3)	375(0.8)	1522(3.3)
Financial year					
	2003-2004	2622(4.4)	1188(4.2)	2227(4.9)	2165(4.7)
	2004-2005	3067(5.2)	1593(5.7)	3090(6.8)	3886(8.5)
	2005-2006	5103(8.6)	2385(8.5)	3830(8.4)	4147(9.0)
	2006-2007	6719(11.3)	2859(10.2)	4458(9.8)	4974(10.8)
	2007-2008	6635(11.2)	3102(11.1)	4958(10.9)	4908(10.7)
	2008-2009	5992(10.1)	2972(10.6)	5001(11.0)	5370(11.7)
	2009-2010	6949(11.7)	3215(11.5)	5148(11.3)	5336(11.6)
	2010-2011	7525(12.7)	3475(12.4)	5243(11.5)	5012(10.9)
	2011-2012	7930(13.4)	3740(13.4)	5811(12.7)	5478(11.9)
	2013-2013	6824(11.5)	3485(12.4)	5855(12.8)	4647(10.1)

IQR=Inter quartile range; SD= Standard deviation; Home care packages (2003-2013); CACP=Community Aged Care Package; EACH=Extended Aged Care at Home; EACHD= Extended Aged Care at Home Dementia; Home care packages (Since 2014); Level1 = Home Care Level 1; Level2 = Home Care Level 2; Level3 = Home Care Level 3; Level4 = Home Care Level 4.

### Characteristics associated with wait time to receive a home care package

Wait time was associated with age, sex, country of birth, whether an individual lived with someone or alone, owned their accommodation, had an activity limitation (communication, domestic assistance, health care tasks, meals, self-care, social and community participation, transportation, and social activity), and health conditions (Table 2). Compared with people who waited 0–30 days (OR, 95%CI), individuals who waited >6 months were more likely to be born overseas (1.07, 1.04-1.10); live with family (1.65, 1.61-1.70) or others (1.36, 1.24-1.49) compared to living alone; from ACT (1.31, 1.17-1.47), NSW (1.52, 1.45-1.6), TAS (1.46, 1.33-1.61),VIC (1.09, 1.04-1.15) compared to being in the state of SA; and have approvals also for residential aged care (1.11, 1.08-1.15), respite care (1.38, 1.33-1.43), transition care program (4.34, 3.94-4.78). Older people were alternatively less likely to wait longer than 6 months if they were male (0.90, 0.87-0.92); lived in retirement villages (0.75, 0.72-0.79) or short-term/ temporary accommodation (0.75, 0.64-0.88) compared to owning a private accommodation; had activity limitations for communication (0.91, 0.88-0.94), domestic assistance (0.85, 0.79-0.92), health care tasks (0.87, 0.85-0.90), meals (0.77, 0.74-0.80), self-care (0.67, 0.65-0.69), social and community participation (0.88, 0.84-0.91), or transport (0.84, 0.8-0.87); had medical health conditions such as cancer (0.85, 0.82-0.88); and geriatric syndromes such as falls (0.89, 0.86-0.93) or delirium (0.65, 0.54-0.78). Similar trends of associations were observed between the individual’s characteristics and whether they waited 31–59 days or 60 days-6 months (Table 2).

### The impact of wait time on mortality

The cohort was followed for 4.0 years (interquartile range (IQR:1.8-7.2)) and 38% were alive at the end of the study period (Supplementary Table 2). Individuals waiting >6 months had an 18% higher risk of death after 2 years of starting their HCP, when contrasted to individuals receiving a HCP within 30 days (adjusted hazard ratio (aHR, 95%CI=1.18, 1.16-1.21)). Those who waited 31–59 days had a 6% higher mortality risk (1.06, 1.03-1.09) after 2.5 years of entry and the risk of mortality for those who waited 2–6 months was a 4% higher (1.04, 1.00-1.08) when compared to those who waited < 30 days after five years (Table 3a, Figure 1a). The risk of mortality following transition into permanent residential aged care for people who waited > than 6 months was 8% higher (1.08, 1.05-1.10) compared to people receiving HCP within 30 days. Similar associations were noted for the groups who waited 2–6 months (1.03, 1.01-1.05) and 31–59 days (1.03, 1.01-1.05) (Table 3b, Figure 1b).

### The impact of wait time on transition to permanent residential aged care

Of the 178,924 older people who had a HCP, 92,987 (52.0%) transitioned to a permanent residential aged care service. The median time (IQR) of transition was 2.8 (1.0-8.0) years. The cumulative incidence of transition, % (95%CI), from a HCP to permanent residential aged care service at 30 days, 3 months, 6 months, 1 year, 2 years and 5 years was 1.0 (1.0-1.0), 6.0 (6.0-6.0), 14.0 (13.0-14.0), 26.0 (26.0-26.0), 42.0 (41.0-42.0) and 65.0 (64.0-65.0), respectively (Supplementary Table 3, Figure 2). After 2 years of stay in a HCP, people who waited > 6 months had 10% (aHR, 95%CI = 1.10, 1.06 -1.13) higher risk of transition to permanent residential aged care. The risk for those who waited for 2–6 months and 31–59 days was higher by 7% after 2 years (1.07, 1.04-1.11) and 4% after one year (1.04, 1.02-1.07) compared to individuals receiving the HCP within a month (Table 3c).

### Sensitivity analyses

HCP entry care level was not an effect modifier for risk of mortality (in both models) and transition into permanent care (data not shown), except in the case of people who were approved for EACHD and waited for >6 months (N=2679) when compared to <=30 days (N=2756). Within this group the risk of mortality after entry into HCP was higher within 2 years (aHR=1.13, 95%CI= 1.03-1.24), which was not observed in the other groups where the risk was only observed 2 years after entry into HCP.

**Figure 1a:**
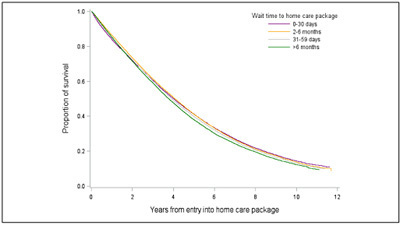
Cumulative survival probability after accessing a home care package by wait time

**Table 2:** Associations of individual characteristics with likelihood of wait time periods (Odds Ratios and 95%CI, 0–30 days as a reference)

**Variables**				**Wait time**			
	**Categories**	**31–59 days**		**2–6 months**		**Over 6 months**	
		**cOR(95%CI)**	**aOR(95%CI)** ^**1**^	**cOR(95%CI)**	**aOR(95%CI)** ^**1**^	**cOR(95%CI)**	**aOR(95%CI)** ^**1**^
Age (year)		1.00(1.00, 1.00)	1.00(1.00, 1.00)	1.00(1.00, 1.00)	1.00(0.99, 1.00)	0.99(0.99, 0.99)	0.99(0.99, 0.99)
Sex	Male vs. Female	0.96(0.93, 0.99)	0.96(0.93, 0.99)	0.92(0.90, 0.95)	0.91(0.88, 0.93)	0.94(0.92, 0.97)	0.90(0.87, 0.92)
Indigenous status	Neither vs. Aboriginal/TSI	2.21(1.91, 2.54)	1.88(1.62, 2.18)	2.03(1.81, 2.27)	1.86(1.64, 2.11)	1.75(1.57, 1.95)	1.46(1.30, 1.63)
Country of birth	Born overseas vs. Australia	0.99(0.96, 1.02)	0.95(0.92, 0.98)	1.02(0.99, 1.04)	1.00(0.98, 1.03)	1.07(1.04, 1.1)	1.07(1.04, 1.10)
Living arrangements							
	Lives with family vs. Lives alone	1.04(1.01, 1.07)	1.03(1.00, 1.06)	1.09(1.06, 1.11)	1.13(1.10, 1.16)	1.45(1.41, 1.48)	1.65(1.61, 1.70)
	Lives with others vs. Lives alone	1.00(0.90, 1.11)	0.99(0.89, 1.10)	1.00(0.92, 1.10)	0.89(0.80, 0.98)	1.26(1.15, 1.37)	1.36(1.24, 1.49)
Remoteness	Other vs. Major city	0.90(0.87, 0.93)	0.92(0.89, 0.95)	0.93(0.91, 0.96)	0.93(0.90, 0.95)	0.96(0.94, 0.99)	1.00(0.98, 1.03)
Usual accommodation							
	Other hospital/ residential vs. Private	0.94(0.82, 1.07)	1.04(0.90, 1.19)	0.98(0.88, 1.10)	0.92(0.82, 1.04)	0.92(0.82, 1.03)	0.97(0.86, 1.09)
	Retirement village vs. private	0.80(0.77, 0.84)	0.83(0.79, 0.87)	0.74(0.71, 0.77)	0.81(0.77, 0.84)	0.67(0.65, 0.70)	0.75(0.72, 0.79)
	Short term/temporary vs. private	0.67(0.57, 0.79)	0.83(0.70, 0.97)	0.50(0.43, 0.58)	0.72(0.62, 0.85)	0.44(0.37, 0.51)	0.75(0.64, 0.88)
State							
	ACT vs. SA	1.30(1.15, 1.47)	1.19(1.05, 1.35)	1.12(1.01, 1.25)	0.92(0.82, 1.03)	1.76(1.59, 1.94)	1.31(1.17, 1.47)
	NSW vs. SA	1.28(1.21, 1.35)	1.20(1.14, 1.27)	1.41(1.35, 1.48)	1.44(1.37, 1.51)	1.55(1.48, 1.63)	1.52(1.45, 1.60)
	NT vs. SA	0.57(0.48, 0.68)	0.85(0.72, 1.01)	0.53(0.46, 0.61)	0.75(0.65, 0.87)	0.62(0.54, 0.71)	0.68(0.59, 0.79)
	QLD vs. SA	0.75(0.71, 0.80)	0.74(0.69, 0.78)	0.50(0.47, 0.52)	0.53(0.50, 0.56)	0.67(0.63, 0.70)	0.70(0.66, 0.74)
	TAS vs. SA	1.21(1.09, 1.34)	1.22(1.10, 1.36)	1.17(1.07, 1.27)	1.51(1.38, 1.65)	1.23(1.13, 1.35)	1.46(1.33, 1.61)
	VIC vs. SA	1.35(1.28, 1.43)	1.34(1.26, 1.42)	1.18(1.12, 1.24)	1.24(1.18, 1.31)	1.16(1.10, 1.22)	1.09(1.04, 1.15)
	WA vs. SA	0.66(0.62, 0.70)	0.63(0.59, 0.67)	0.45(0.43, 0.48)	0.42(0.40, 0.44)	0.65(0.61, 0.68)	0.65(0.62, 0.69)
Financial year							
	2004-2005 vs. 2003-2004	1.15(1.05, 1.26)	1.29(1.17, 1.42)	1.19(1.10, 1.28)	1.24(1.15, 1.34)	1.53(1.42, 1.65)	1.59(1.47, 1.72)
	2005-2006 vs. 2003-2004	1.03(0.95, 1.12)	1.28(1.17, 1.40)	0.88(0.82, 0.95)	1.17(1.08, 1.27)	0.98(0.92, 1.06)	1.28(1.19, 1.39)
	2006-2007 vs. 2003-2004	0.94(0.87, 1.02)	1.31(1.18, 1.46)	0.78(0.73, 0.84)	1.08(0.98, 1.18)	0.90(0.84, 0.96)	1.33(1.21, 1.46)
	2007-2008 vs. 2003-2004	1.03(0.95, 1.12)	1.48(1.31, 1.66)	0.88(0.82, 0.94)	1.21(1.09, 1.34)	0.90(0.84, 0.96)	1.41(1.28, 1.56)
	2008-2009 vs. 2003-2004	1.09(1.01, 1.19)	1.59(1.41, 1.78)	0.98(0.92, 1.05)	1.43(1.29, 1.58)	1.09(1.01, 1.16)	1.75(1.58, 1.93)
	2009-2010 vs. 2003-2004	1.02(0.94, 1.11)	1.48(1.32, 1.67)	0.87(0.82, 0.93)	1.32(1.19, 1.46)	0.93(0.87, 0.99)	1.55(1.40, 1.71)
	2010-2011 vs. 2003-2004	1.02(0.94, 1.10)	1.47(1.31, 1.66)	0.82(0.77, 0.88)	1.22(1.10, 1.35)	0.81(0.75, 0.86)	1.32(1.19, 1.46)
	2011-2012 vs. 2003-2004	1.04(0.96, 1.13)	1.49(1.32, 1.67)	0.86(0.81, 0.92)	1.27(1.15, 1.41)	0.84(0.78, 0.89)	1.37(1.24, 1.52)
	2012-2013 vs. 2003-2004	1.13(1.04, 1.22)	1.64(1.46, 1.84)	1.01(0.95, 1.08)	1.47(1.33, 1.63)	0.82(0.77, 0.88)	1.38(1.24, 1.53)
Activity limitation							
Communication	Yes vs. No	0.96(0.92, 0.99)	1.00(0.96, 1.04)	0.96(0.92, 0.99)	1.01(0.97, 1.05)	0.89(0.86, 0.92)	0.91(0.88, 0.94)
Domestic assistance	Yes vs. No	0.93(0.85, 1.02)	1.04(0.95, 1.15)	0.77(0.71, 0.82)	0.92(0.84, 0.99)	0.68(0.63, 0.73)	0.85(0.79, 0.92)
Health care tasks	Yes vs. No	1.02(0.98, 1.05)	0.99(0.96, 1.03)	0.98(0.95, 1.01)	0.99(0.96, 1.02)	0.80(0.78, 0.82)	0.87(0.85, 0.90)
Meals	Yes vs. No	0.91(0.87, 0.95)	0.91(0.87, 0.95)	0.82(0.79, 0.85)	0.92(0.88, 0.95)	0.69(0.66, 0.71)	0.77(0.74, 0.80)
Movement activities	Yes vs. No	1.01(0.97, 1.05)	1.04(1.00, 1.09)	1.05(1.02, 1.08)	1.06(1.02, 1.10)	1.02(0.98, 1.05)	1.05(1.01, 1.09)
Self-care	Yes vs. No	0.87(0.85, 0.90)	0.92(0.89, 0.95)	0.80(0.78, 0.82)	0.83(0.80, 0.85)	0.65(0.63, 0.67)	0.67(0.65, 0.69)
Social and community participation	Yes vs. No	0.95(0.91, 0.99)	0.98(0.93, 1.02)	0.86(0.84, 0.89)	0.97(0.93, 1.00)	0.73(0.71, 0.76)	0.88(0.84, 0.91)
Transport	Yes vs. No	0.90(0.86, 0.95)	0.93(0.88, 0.99)	0.85(0.81, 0.88)	0.90(0.86, 0.94)	0.71(0.68, 0.74)	0.84(0.80, 0.87)
Moving around places at or away from home	Yes vs. No	0.97(0.94, 1.00)	1.02(0.99, 1.06)	0.98(0.95, 1.00)	0.99(0.97, 1.02)	0.90(0.88, 0.93)	0.99(0.96, 1.02)
Approvals							
Permanent Care	Yes vs. No	1.02(0.99, 1.05)	1.06(1.03, 1.10)	0.94(0.92, 0.97)	1.07(1.04, 1.11)	1.03(1.01, 1.06)	1.11(1.08, 1.15)
Respite Care	Yes vs. No	1.19(1.15, 1.23)	1.14(1.10, 1.19)	1.1(1.07, 1.13)	1.12(1.08, 1.16)	1.31(1.27, 1.35)	1.38(1.33, 1.43)
Transition care	Yes vs. No	2.20(1.98, 2.45)	2.82(2.52, 3.14)	5.96(5.48, 6.49)	8.02(7.33, 8.78)	3.04(2.77, 3.33)	4.34(3.94, 4.78)

**Table 3a:** Associations of wait time for home care package and the risk of mortality after entry into the home care package (Hazard Ratio and 95%CI, 0–30 days as a reference)

**Wait time**	**31–59 days vs. 0–30 days** ^**1**^		**2–6 months vs. 0–30 days** ^**2**^		**Over 6 months vs. 0–30 days** ^**3**^	
**Follow-up time after entry**	**cHR(95%CI)**	**aHR(95%CI)** ^**4**^	**cHR(95%CI)**	**aHR(95%CI)** ^**4**^	**cHR(95%CI)**	**aHR(95%CI)** ^**4**^
Within 2.5 years	0.92(0.89, 0.94)	0.93(0.91, 0.95) ***				
After 2.5 years	1.06(1.03, 1.09)	1.06(1.03, 1.09) ***				
Within 5 years			0.95(0.94, 0.97)	0.98(0.96, 0.99) ***		
After 5 years			1.04(1.01, 1.08)	1.04(1.00, 1.08) ***		
Within 2 years					0.59(0.58, 0.61)	0.63(0.62, 0.65) ***
After 2 years					1.11(1.09, 1.13)	1.18(1.16, 1.21) ***

*** P<0.0001. cHR=crude Hazard Ratio. aHR=adjusted Hazard Ratio; ^1^Model N=86956 (missing data=424/87380, 0.5%); ^2^Model N=104439 (missing data=548/104987, 0.5%); ^3^Model N=104550 (missing data= 739/105289, 0.7%); ^4^All models adjusted for covariates associated with wait time: age, sex, country of birth, indigenous status, living arrangements, remoteness, usual accommodation, activity limitations (communication, domestic assistance, health care tasks, meals, movement activities, self-care, social and community participation, transport, moving around places at or away from home), approval for (permanent, respite, transition care), health conditions (hypertension, falls, arthritis, diseases of the skin and subcutaneous tissue, cancer, delirium, dementia, depression, diabetes, fracture).

**Table 3b:** Associations of wait time for home care package and the risk of mortality after entry into permanent residential aged care (Hazard Ratio and 95%CI, 0–30 days as a reference)

**Wait time**	**cHR(95%CI)**	**aHR(95%CI)** ^**1**^
31–59 days vs. 0–30 days	1.01 (0.99, 1.04)	1.03 (1.01, 1.05) ***
2–6 months vs. 0–30 days	1.01 (0.99, 1.03)	1.03 (1.01, 1.05) ***
Over 6 months vs. 0–30 days	1.03 (1.01, 1.05)	1.08 (1.05, 1.10) ***

*** P<0.0001; cHR: crude Hazard Ratio, aHR: adjusted Hazard Ratio; 1. Model N=92232 (missing data=755/92987, 0.8%). Model adjusted for covariates associated with wait time: age, sex, country of birth, indigenous status, living arrangements, remoteness, usual accommodation, activity limitations (communication, domestic assistance, health care tasks, meals, movement activities, self-care, social and community participation, transport, moving around places at or away from home), approval for (permanent, respite, transition care), health conditions (hypertension, falls, arthritis, diseases of the skin and subcutaneous tissue, cancer, delirium, dementia, depression, diabetes, fracture).

**Figure 1b:**
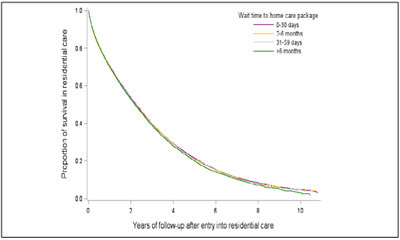
Cumulative survival probability after entry into permanent residential aged care services by wait time for home care packages

## Discussion

The key findings from this research were that although almost 40% of those commencing with a HCP were still alive in this study at four years, when compared to those waiting less than 30 days for a HCP, those waiting more than six months had a higher risk for mortality and transition into permanent residential aged care services after two years. Additionally, for those who transitioned into permanent residential aged care services, mortality risk was higher for those waiting more than six months than those waiting less than 30 days and this was noticable after two years as well. By 2 years, there was also an increased risk of transition to permanent residential aged care for those waiting 30 days or longer with, the highest risk seen for those waiting more than six months. What is not known is if interventions to reduce the waiting time including the use of interim strategies such as lower level of packages or short term services especially for those waiting more than six months, might mitigate some of the detrimental risks seen and result in better survival outcomes and reduced future reliance on permanent residential aged care services. The use of registry data such as this to evaluate the impact of policy or service model changes over time is useful.

It is very likely that a large proportion of those who were assessed as requiring HCPs were frail. We have previously reported that over a period of up to 4.5 years, almost half of the community dwelling older people in one South Australian cohort study worsened either through mortality or progression of their frailty status (13). Eleven percent of pre-frail and 30% of frail older people in that study had died by end of the follow-up period (13). The recipients of HCP in this current study were more likely to be frailer than the participants in the longitudinal cohort study and this would account for the almost 60% mortality rate seen. In population groups where frailty is common, integrated health and aged care services is an imperative and therefore, collaboration with general practitioners and other primary care providers in the achievement of coordinated health and aged care is vital if needs are to be better met within existing resources for this population group. Better integration of care between the health and aged care systems with the consumer as the focus could improve outcomes for this population group.

**Table 3c:** Risk of transition from a home care package to permanent residential aged care by home care package wait time (Hazard Ratio and 95%CI, 0–30 days as a reference)

**Wait time**	**31–59 days vs. 0–30 days** ^**1**^		**2–6 months vs. 0–30 days** ^**2**^		**Over 6 months vs. 0–30 days** ^**3**^	
	**(N=87380, N-adjusted= 86956)**		**(N=104987, N-adjusted= 104439)**		**(N=105289, N-adjusted= 104550)**	
**Follow-up time after entry**	**cHR(95%CI)**	**aHR(95%CI)** ^**4**^	**cHR(95%CI)**	**aHR(95%CI)** ^**4**^	**cHR(95%CI)**	**aHR(95%CI)** ^**4**^
Within 1 year	0.98(0.95, 1.01)	1.00(0.97, 1.03) ***				
After 1 year	1.07(1.04, 1.09)	1.04(1.02, 1.07) ***				
Within 2 years			0.87(0.85, 0.89)	0.90(0.88, 0.92) ***		
After 2 years			1.09(1.06, 1.12)	1.07(1.04, 1.11) ***		
Within 2 years					0.74(0.73, 0.76)	0.79(0.77, 0.80) ***
After 2 years					1.12(1.08, 1.15)	1.10(1.06, 1.13) ***

*** P<0.0001; cHR: crude Hazard Ratio, aHR: adjusted Hazard Ratio; 1. Model N=86956 (missing data=424/87380, 0.5%); ^2^Model N=104439 (missing data=548/104987, 0.5%); ^3^Model N=104550 (missing data= 739/105289, 0.7%); 4. All models adjusted for covariates associated with wait time: age, sex, country of birth, indigenous status, living arrangements, remoteness, usual accommodation, activity limitations (communication, domestic assistance, health care tasks, meals, movement activities, self-care, social and community participation, transport, moving around places at or away from home), approval for (permanent, respite, transition care), health conditions (hypertension, falls, arthritis, diseases of the skin and subcutaneous tissue, cancer, delirium, dementia, depression, diabetes, fracture).

**Figure 2:**
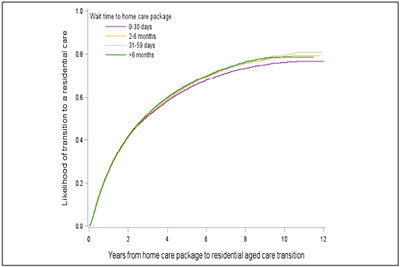
Cumulative incidence of transition into permanent residential aged care services by wait time for home care packages

While the effects we studied (i.e. mortality and entry into permanent care) were seen 2 years after starting a HCP, the lack of timely access to care is likely to have immediate consequences on older people and their carers. Over time, unmet needs may contribute to deterioration in wellbeing with likely increase in the risk of mortality, frailty and disability as well as reduce well-being. The discussion around unmet aged care needs is not confined to Australia and many jurisdictions around the world are planning and implementating new policies or models of care to better meet the changing needs of our society at large. For example, it was recently highlighted that of the 12 million older Americans requiring assistance with household tasks for health and functioning reasons, at least 2 million reported at least one unmet need including going without groceries or a hot meal(14). Poor nutritional health is associated with poor health outcomes such as falls, hospitalisation and premature residential care placement(15, 16). Accordingly, a recent prospective cohort study of community living, older disabled Americans, found that the incidence rate of emergency department admissions was higher for those with unmet needs, especially as a result of falls and other injuries (17). Whilst some might argue that we cannot afford to meet the gap in services, the reality is that there are likely to be knock on costs to older people and their informal carers as a direct result of poor health as well as increased costs to hospital systems as the older person begins to rely on more expensive health services such as hospital care.

When compared to those waiting for less than 30 days, those waiting for more than six months were more likely to be from culturally and linguistically diverse backgrounds, be living with family or others as opposed to living alone and be approved for alternative levels of care such as respite, permanent placement or transition care program. Older people from ethnic minorities and so from culturally and linguistically diverse backgrounds may refuse HCPs because of feelings of shame when receiving care from a non-family member, gender sensitivity and difficulties arising from the clash between western health practices with their cultural norms (18). There could also be an erroneous perception that those living with others will have their care needs met because they have informal carers. In a recent study, whilst participants had expectations for informal care prior to having a need, approximately 30% did not have needs met (19). Of this group with unmet needs, almost 30% were living without any of the care they required being met.

In this current study also, men, those from accommodation such as retirement villages or short-term accommodation, those with geriatric syndromes such as falls or delirium or illnesses like cancer and people with difficulties with personal activities of daily living, communication, transportation and community participation were less likely to wait six months or more when compared to those waiting less than 30 days. Health conditions such as delirium, falls and cancer increase an older person’s risk of mortality or residential age care placement (20). Older people with higher activity of daily living needs may overwhelm caregiver capacity sooner thus resulting in an earlier reliance on HCPs or transition residential aged care services (19). Whilst at times delays to access might be due to consumer preference, it is also possible that those who are more ill as well as those with more visible needs such as difficulties performing activities of daily living may be prioritised over others for HCPs.

Given the high mortality rate where 60% had passed away by four years, the encouragement of advance care planning, especially prior to the onset of dementia or at the time of diagnosis should be encouraged. Sadly, there may be a gap in this aspect of care. A 2012 survey of providers of home care packages across Australia revealed that at most one third had policies or procedures with less than half of case managers trained (21). To add to this shortcoming, a more recent study described that advance care directives were initiated for only 65% of clients and even when completed was of varying quality (22). The need for skills would apply equally to the management of other geriatric syndromes such as dementia, delirium, falls, frailty, malnutrition and polypharmacy. It needs to also be noted that there have been policy initiatives around advance care directives and training of staff and possibly, things may be improved. The solution therefore is not as simple as merely funding more HCPs but instead it needs to be coupled with other strategies including capacity building through the delivery of a skilled aged care workforce. The Australian aged care taskforce recently highlighted key themes that required consideration including the underestimated value of the role of personal care workers, the lack of a realistic career progression for workers, nursing burnout from being pulled in many directions, a misalignment between competencies and skills and the current education framework, attraction and retention difficulties perceived to relate in part to remuneration and difficulties balancing between need for clinical and managerial skills within the sector (23).

An important strength of this study was its comprehensiveness. This study fully captured people who underwent an ACAT assessment during the study period nationally using a systematic data capture system that relied on skilled assessors operating as per a standardized system nationally. The results of the study are therefore generalizable to people being assessed for HCPs across Australia with relevance to similar population groups in other countries with publicly funded long-term care systems. However, our study has limitations due to its observational and retrospective nature. Because this is an observational study causation cannot be implied from our estimates. Additionally, while we attempted to adjust our estimates to all confounding variables available to us, residual confounding is still a possibility and we recognise that important variables such as acuity of illness, consumer choice, mix of packages by region and provider prioritisation about entry into HCP were not captured and could influence both the wait times as well as outcomes. The study also includes small but statistically significant associations that may be of limited clinical value but are expected with large sample sizes.

To summarise, the wait time for HCPs is associated with reduced longevity even after receiving services and noticeable after two years in those waiting longer than six months. Additionally, the increased likelihood of permanent residential aged care service placement is similarly noticeable after two years for those waiting six months or longer for a HCP. While the whole situation may be more complex, meeting the needs of consumer choice through transparency and an increase in a number of HCPs would seem to be a step in the right direction. Other strategies going forward may include better targeting of HCPs to those most at need, better integration between health and aged care services to keep people healthier for longer as well as addressing the workforce issues ensuring quality of care for recipients. Doing nothing comes at an increased personal cost to older people and those that care for them and, as this study indicates, for the society more generally.

*Conflict of Interest:* Mr Richard Hearn is the Chief Executive Officer, Ms Sue McKechnie is the Executive Manager Community Services and Professor Renuka Visvanathan is a Board Member of Resthaven Inc., a not for profit aged care community service associated with the Uniting Church in Australia but separately incorporated, financially independent and a charitable Public Benevolent Institution. Ms Jane Mussared is the Chief Executive Officer of Council on the Ageing South Australia, a peak body for 630,000 older people in South Australia providing a platform to ensure that older South Australians are part of the decision-making by government and industry.

*Acknowledgment:* We would like to acknowledge the Healthy Ageing Research Consortium Investigator Team and the ROSA’s South Australian Health and Medical Research Institute (SAHMRI) Research Team for ensuring the success of the ROSA and support with this study. We also acknowledge the South Australian Government who provide us with support (2017-2021) through the Department for Industry and Skills, and the Australian Institute of Health and Welfare (AIHW) for the provision of the raw data used in the ROSA.

*Funding:* South Australian Government Department for Industry and Skills, National Health and Medical Research Council Centre of Research Excellence Scheme (APP 1102208), Medical Research Future Fund (MRFF) Rapid Applied Research Translation Program.

*Ethical Standards:* Human research ethics committee approval for this study was received before study commencement from the University of South Australia ethics committee-ID 200489. The research has been conducted in accordance with the National Health and Medical Research Council National Statement on Ethical Conduct in Human Research.

## Electronic supplementary material

Supplementary Table 1. Characteristics of individuals by quartiles of wait time for home care packages, 2003–2013

Supplementary material, approximately 41.9 KB.

Supplementary material, approximately 158 KB.
