# Supplementary material for: Prolonged Wait Time Prior to Entry to Home Care Packages Increases the Risk of Mortality and Transition to Permanent Residential Aged Care Services: Findings from the Registry of Older South Australians (ROSA)
Source: J Nutr Health Aging. 2018 Dec 4;23(3):271–80. doi: 10.1007/s12603-018-1145-y (PMC6399871; doi:10.1007/s12603-018-1145-y)
Supplement: Supplementary file 1 — Supplementary Table 1. Characteristics of individuals by quartiles of wait time for home care packages, 2003–2013 [file mmc1.docx]

**Supplementary Table 1. Characteristics of individuals by quartiles of wait time for home care packages, 2003-2013**

| Variables | | Wait time | | | |
| --- | --- | --- | --- | --- | --- |
|  |  | **0-30 days** | **31-59 days** | **2-6 months** | **Over 6 months** |
| Total cases; N (%) | 178924 (100) | 59366(33.2) | 28014(15.7) | 45621(25.5) | 45923(25.6) |
| Indigenous Status |  |  |  |  |  |
|  | Aboriginal/TS Islander | 1083(1.8) | 234(0.8) | 415(0.9) | 482(1.0) |
|  | Neither | 58156(98) | 27737(99) | 45139(98.9) | 45399(98.9) |
|  | Missing | 127(0.2) | 43(0.2) | 67(0.1) | 42(0.1) |
| English proficiency index | |  |  |  |  |
|  | EP0 | 39646(66.8) | 18795(67.1) | 30292(66.4) | 29967(65.3) |
|  | EP1 | 8621(14.5) | 3676(13.1) | 5734(12.6) | 5819(12.7) |
|  | EP2 | 4177(7.0) | 1974(7.0) | 3263(7.2) | 3393(7.4) |
|  | EP3 | 6578(11.1) | 3411(12.2) | 6059(13.3) | 6466(14.1) |
|  | EP4 | 333(0.6) | 155(0.6) | 266(0.6) | 275(0.6) |
|  | Missing | 11(<0.1) | 3(<0.1) | 7(<0.1) | 3(<0.1) |
| Living arrangements | |  |  |  |  |
|  | Lives alone | 30098(50.7) | 13957(49.8) | 22187(48.6) | 19060(41.5) |
|  | Lives with family | 27669(46.6) | 13311(47.5) | 22131(48.5) | 25377(55.3) |
|  | Lives with others | 1137(1.9) | 527(1.9) | 842(1.8) | 904(2.0) |
|  | Not applicable | 123(0.2) | 41(0.1) | 102(0.2) | 73(0.2) |
|  | Missing | 339(0.6) | 178(0.6) | 359(0.8) | 509(1.1) |
| Priority |  |  |  |  |  |
|  | Within 48 hours | 2478(4.2) | 1035(3.7) | 1760(3.9) | 1650(3.6) |
|  | Between 3 and 14 days | 27897(47) | 11930(42.6) | 19689(43.2) | 18757(40.8) |
|  | More than14 days | 28844(48.6) | 14967(53.4) | 24042(52.7) | 25371(55.2) |
|  | Missing | 147(0.2) | 82(0.3) | 130(0.3) | 145(0.3) |
| State |  |  |  |  |  |
|  | ACT | 853(1.4) | 506(1.8) | 785(1.7) | 1105(2.4) |
|  | NSW | 15376(25.9) | 8970(32) | 17798(39.0) | 17545(38.2) |
|  | NT | 697(1.2) | 181(0.6) | 301(0.7) | 318(0.7) |
|  | QLD | 13706(23.1) | 4681(16.7) | 5602(12.3) | 6728(14.7) |
|  | SA | 4954(8.3) | 2259(8.1) | 4067(8.9) | 3646(7.9) |
|  | TAS | 1335(2.2) | 734(2.6) | 1280(2.8) | 1210(2.6) |
|  | VIC | 12444(21.0) | 7669(27.4) | 12057(26.4) | 10611(23.1) |
|  | WA | 10001(16.8) | 3014(10.8) | 3731(8.2) | 4760(10.4) |
| Usual accommodation |  |  |  |  |  |
|  | Private (owned or rental) | 50323(84.8) | 24428(87.2) | 40140(88) | 40638(88.5) |
|  | Short term temporary supported | 599(1.0) | 195(0.7) | 240(0.5) | 212(0.5) |
|  | Retirement village independent unit | 7479(12.6) | 2907(10.4) | 4404(9.7) | 4071(8.9) |
|  | Hospital/residential/other | 704(1.2) | 321(1.1) | 550(1.2) | 525(1.1) |
|  | Missing | 477(1.0) | 261(0.4) | 287(0.6) | 163(0.6) |
| *Health conditions* |  |  |  |  |  |
| Deafness/hearing loss | |  |  |  |  |
|  | No | 50310(84.7) | 23657(84.4) | 38454(84.3) | 39209(85.4) |
|  | Yes | 9056(15.3) | 4357(15.6) | 7167(15.7) | 6714(14.6) |
| Hypertension |  |  |  |  |  |
|  | No | 32375(54.5) | 15189(54.2) | 24559(53.8) | 25339(55.2) |
|  | Yes | 26991(45.5) | 12825(45.8) | 21062(46.2) | 20584(44.8) |
| Chronic lower respiratory diseases | |  |  |  |  |
|  | No | 49449(83.3) | 23392(83.5) | 38500(84.4) | 38947(84.8) |
|  | Yes | 9917(16.7) | 4622(16.5) | 7121(15.6) | 6976(15.2) |
| Osteoporosis |  |  |  |  |  |
|  | No | 48703(82) | 22828(81.5) | 37123(81.4) | 37275(81.2) |
|  | Yes | 10663(18) | 5186(18.5) | 8498(18.6) | 8648(18.8) |
| Kidney & urinary system disorders | |  |  |  |  |
|  | No | 54484(91.8) | 25681(91.7) | 41880(91.8) | 42498(92.5) |
|  | Yes | 4882(8.2) | 2333(8.3) | 3741(8.2) | 3425(7.5) |
| Falls (unknown aetiology) | |  |  |  |  |
|  | No | 50193(84.5) | 23622(84.3) | 38270(83.9) | 39326(85.6) |
|  | Yes | 9173(15.5) | 4392(15.7) | 7351(16.1) | 6597(14.4) |
| Arthritis |  |  |  |  |  |
|  | No | 34537(58.2) | 16088(57.4) | 26316(57.7) | 26338(57.4) |
|  | Yes | 24829(41.8) | 11926(42.6) | 19305(42.3) | 19585(42.6) |
| Diseases of the skin and subcutaneous tissue | | |  |  |  |
|  | No | 56738(95.6) | 26685(95.3) | 43442(95.2) | 43978(95.8) |
|  | Yes | 2628(4.4) | 1329(4.7) | 2179(4.8) | 1945(4.2) |
| Cancer |  |  |  |  |  |
|  | No | 49445(83.3) | 23443(83.7) | 38647(84.7) | 39250(85.5) |
|  | Yes | 9921(16.7) | 4571(16.3) | 6974(15.3) | 6673(14.5) |
| Cardiovascular diseases | |  |  |  |  |
|  | Yes | 51386(86.6) | 24050(85.8) | 39023(85.5) | 39269(85.5) |
|  | No | 7980(13.4) | 3964(14.2) | 6598(14.5) | 6654(14.5) |
| Delirium |  |  |  |  |  |
|  | No | 59045(99.5) | 27872(99.5) | 45277(99.2) | 45715(99.5) |
|  | Yes | 321(0.5) | 142(0.5) | 344(0.8) | 208(0.5) |
| Dementia |  |  |  |  |  |
|  | No | 46427(78.2) | 21442(76.5) | 34899(76.5) | 35690(77.7) |
|  | Yes | 12939(21.8) | 6572(23.5) | 10722(23.5) | 10233(22.3) |
| Depression |  |  |  |  |  |
|  | No | 50029(84.3) | 23355(83.4) | 38258(83.9) | 38602(84.1) |
|  | Yes | 9337(15.7) | 4659(16.6) | 7363(16.1) | 7321(15.9) |
| Diabetes |  |  |  |  |  |
|  | No | 47488(80.0) | 22311(79.6) | 36362(79.7) | 36782(80.1) |
|  | Yes | 11878(20.0) | 5703(20.4) | 9259(20.3) | 9141(19.9) |
| Eye diseases |  |  |  |  |  |
|  | No | 44123(74.3) | 20685(73.8) | 33484(73.4) | 33813(73.6) |
|  | Yes | 15243(25.7) | 7329(26.2) | 12137(26.6) | 12110(26.4) |
| Fractures |  |  |  |  |  |
|  | No | 53593(90.3) | 25243(90.1) | 40641(89.1) | 41384(90.1) |
|  | Yes | 5773(9.7) | 2771(9.9) | 4980(10.9) | 4539(9.9) |
| Incontinence |  |  |  |  |  |
|  | No | 53146(89.5) | 24858(88.7) | 40442(88.6) | 41169(89.6) |
|  | Yes | 6220(10.5) | 3156(11.3) | 5179(11.4) | 4754(10.4) |
| Malnutrition |  |  |  |  |  |
|  | No | 58182(98.0) | 27419(97.9) | 44689(98.0) | 45067(98.1) |
|  | Yes | 1184(2.0) | 595(2.1) | 932(2.0) | 856(1.9) |
| *Activity limitations* | |  |  |  |  |
| Communication |  |  |  |  |  |
|  | No | 49963(84.2) | 23741(84.7) | 38660(84.7) | 39298(85.6) |
|  | Yes | 9352(15.8) | 4247(15.2) | 6921(15.2) | 6573(14.3) |
|  | Missing | 51(0.1) | 26(0.1) | 40(0.1) | 52(0.1) |
| Domestic assistance | |  |  |  |  |
|  | No | 1441(2.4) | 728(2.6) | 1436(3.1) | 1616(3.5) |
|  | Yes | 57874(97.5) | 27260(97.3) | 44145(96.8) | 44255(96.4) |
|  | Missing | 51(0.1) | 26(0.1) | 40(0.1) | 52(0.1) |
| Health care tasks |  |  |  |  |  |
|  | No | 15101(25.4) | 7044(25.1) | 11768(25.8) | 13749(29.9) |
|  | Yes | 44214(74.5) | 20944(74.8) | 33813(74.1) | 32122(69.9) |
|  | Missing | 51(0.1) | 26(0.1) | 40(0.1) | 52(0.1) |
| Home maintenance | |  |  |  |  |
|  | No | 13097(22.1) | 6242(22.3) | 10515(23.0) | 10497(22.9) |
|  | Yes | 46218(77.9) | 21746(77.6) | 35066(76.9) | 35374(77.0) |
|  | Missing | 51(0.1) | 26(0.1) | 40(0.1) | 52(0.1) |
| Meals |  |  |  |  |  |
|  | No | 7864(13.2) | 4033(14.4) | 7146(15.7) | 8362(18.2) |
|  | Yes | 51451(86.7) | 23955(85.5) | 38435(84.2) | 37509(81.7) |
|  | Missing | 51(0.1) | 26(0.1) | 40(0.1) | 52(0.1) |
| Movement activities | |  |  |  |  |
|  | No | 49944(84.1) | 23520(84.0) | 38082(83.5) | 38527(83.9) |
|  | Yes | 9371(15.8) | 4468(15.9) | 7499(16.4) | 7344(16.0) |
|  | Missing | 51(0.1) | 26(0.1) | 40(0.1) | 52(0.1) |
| Self-care |  |  |  |  |  |
|  | No | 20340(34.3) | 10470(37.4) | 18054(39.6) | 20412(44.4) |
|  | Yes | 38975(65.7) | 17518(62.5) | 27527(60.3) | 25459(55.4) |
|  | Missing | 51(0.1) | 26(0.1) | 40(0.1) | 52(0.1) |
| Social and community participation | |  |  |  |  |
|  | No | 8241(13.9) | 4065(14.5) | 7168(15.7) | 8269(18) |
|  | Yes | 51074(86.0) | 23923(85.4) | 38413(84.2) | 37602(81.9) |
|  | Missing | 51(0.1) | 26(0.1) | 40(0.1) | 52(0.1) |
| Transport |  |  |  |  |  |
|  | No | 4750(8.0) | 2463(8.8) | 4256(9.3) | 4995(10.9) |
|  | Yes | 54565(91.9) | 25525(91.1) | 41325(90.6) | 40876(89.0) |
|  | Missing | 51(0.1) | 26(0.1) | 40(0.1) | 52(0.1) |
| Moving around places at or away from home | | |  |  |  |
|  | No | 27151(45.7) | 13037(46.5) | 21149(46.4) | 22139(48.2) |
|  | Yes | 32164(54.2) | 14951(53.4) | 24432(53.6) | 23732(51.7) |
|  | Missing | 51(0.1) | 26(0.1) | 40(0.1) | 52(0.1) |
| Other |  |  |  |  |  |
|  | No | 56277(94.8) | 26371(94.1) | 42429(93.0) | 42739(93.1) |
|  | Yes | 3038(5.1) | 1617(5.8) | 3152(6.9) | 3132(6.8) |
|  | Missing | 51(0.1) | 26(0.1) | 40(0.1) | 52(0.1) |
| None |  |  |  |  |  |
|  | No | 59241(99.8) | 27946(99.8) | 45496(99.7) | 45751(99.6) |
|  | Yes | 74(0.1) | 42(0.1) | 85(0.2) | 120(0.3) |
|  | Missing | 51(0.1) | 26(0.1) | 40(0.1) | 52(0.1) |
| *Assessor professions* | |  |  |  |  |
| Medical practitioners | |  |  |  |  |
|  | No | 35072(59.1) | 16663(59.5) | 26821(58.8) | 27253(59.3) |
|  | Yes | 23747(40.0) | 11077(39.5) | 18248(40.0) | 18091(39.4) |
|  | Missing | 547(0.9) | 274(1.0) | 552(1.2) | 579(1.3) |
| Nursing professionals | |  |  |  |  |
|  | No | 15539(26.2) | 7307(26.1) | 11933(26.2) | 13134(28.6) |
|  | Yes | 43280(72.9) | 20433(72.9) | 33136(72.6) | 32210(70.1) |
|  | Missing | 547(0.9) | 274(1.0) | 552(1.2) | 579(1.3) |
| Health professionals | |  |  |  |  |
|  | No | 32088(54.1) | 15152(54.1) | 24208(53.1) | 25545(55.6) |
|  | Yes | 26731(45) | 12588(44.9) | 20861(45.7) | 19799(43.1) |
|  | Missing | 547(0.9) | 274(1.0) | 552(1.2) | 579(1.3) |
| Social welfare professionals | |  |  |  |  |
|  | No | 30801(51.9) | 14841(53.0) | 23398(51.3) | 24242(52.8) |
|  | Yes | 28018(47.2) | 12899(46.0) | 21671(47.5) | 21102(46.0) |
|  | Missing | 547(0.9) | 274(1.0) | 552(1.2) | 579(1.3) |
| Not stated |  |  |  |  |  |
|  | No | 58819(99.1) | 27737(99.0) | 45066(98.8) | 45341(98.7) |
|  | Yes | 547(0.9) | 277(1.0) | 555(1.2) | 582(1.3) |
| *Other Service Approvals* |  |  |  |  |  |
| Emergency care |  |  |  |  |  |
|  | No | 59272(99.8) | 27979(99.9) | 45573(99.9) | 45872(99.9) |
|  | Yes | 94(0.2) | 35(0.1) | 48(0.1) | 51(0.1) |
| Permanent residential care |  |  |  |  |  |
|  | No | 29847(50.3) | 13924(49.7) | 23585(51.7) | 22716(49.5) |
|  | Yes | 29519(49.7) | 14090(50.3) | 22036(48.3) | 23206(50.5) |
| Respite care |  |  |  |  |  |
|  | No | 14121(23.8) | 5817(20.8) | 10097(22.1) | 8846(19.3) |
|  | Yes | 45245(76.2) | 22197(79.2) | 35524(77.9) | 37076(80.7) |
| Transition care |  |  |  |  |  |
|  | No | 58684(98.9) | 27310(97.5) | 42715(93.7) | 44426(96.7) |
|  | Yes | 682(1.1) | 704(2.5) | 2906(6.4) | 1497(3.3) |
| Carer Availability |  |  |  |  |  |
|  | Has carer | 47543(80.1) | 23165(82.7) | 37316(81.8) | 37740(82.2) |
|  | Has no carer | 11212(18.9) | 4558(16.3) | 7708(16.9) | 7488(16.3) |
|  | Not applicable | 244(0.4) | 100(0.4) | 234(0.5) | 238(0.5) |
|  | Missing | 367(0.6) | 191(0.7) | 363(0.8) | 457(1) |
| Carer Co-residency Status |  |  |  |  |  |
|  | Co-resident Carer | 21775(36.7) | 10928(39) | 18281(40.1) | 21192(46.1) |
|  | Non-resident Carer | 25728(43.3) | 12228(43.6) | 18972(41.6) | 16509(35.9) |
|  | Not applicable | 11120(18.7) | 4497(16.1) | 7754(17) | 7458(16.2) |
|  | Missing | 743(1.3) | 361(1.3) | 614(1.3) | 764(1.7) |
| Carer relation |  |  |  |  |  |
|  | Friend/neighbour | 2145(3.6) | 1024(3.7) | 1579(3.5) | 1284(2.8) |
|  | Not applicable | 11508(19.4) | 4672(16.7) | 7989(17.5) | 7765(16.9) |
|  | Other relative | 918(1.5) | 417(1.5) | 648(1.4) | 519(1.1) |
|  | Parent | 76(0.1) | 34(0.1) | 48(0.1) | 70(0.2) |
|  | Son in law or daughter in law | 1293(2.2) | 590(2.1) | 892(2) | 829(1.8) |
|  | Son or daughter | 25451(42.9) | 12242(43.7) | 19281(42.3) | 17914(39) |
|  | Spouse partner | 14810(24.9) | 7428(26.5) | 12693(27.8) | 15199(33.1) |
|  | Missing | 3165(5.3) | 1607(5.7) | 2491(5.5) | 2343(5.1) |
| Carer sex |  |  |  |  |  |
|  | Female | 28179(47.5) | 13856(49.5) | 22043(48.3) | 22378(48.7) |
|  | Male | 16514(27.8) | 7879(28.1) | 13098(28.7) | 13437(29.3) |
|  | Not applicable | 11508(19.4) | 4672(16.7) | 7989(17.5) | 7765(16.9) |
|  | Missing | 3165(5.3) | 1607(5.7) | 2491(5.5) | 2343(5.1) |
| *Current Assistance* |  |  |  |  |  |
| Communication |  |  |  |  |  |
|  | No | 50933(85.8) | 23931(85.4) | 38511(84.4) | 38577(84) |
|  | Yes | 8227(13.9) | 3978(14.2) | 6890(15.1) | 7138(15.5) |
|  | Missing | 206(0.3) | 105(0.4) | 220(0.5) | 208(0.5) |
| Domestic assistance |  |  |  |  |  |
|  | No | 6457(10.9) | 2793(10) | 4964(10.9) | 4637(10.1) |
|  | Yes | 52703(88.8) | 25116(89.7) | 40437(88.6) | 41078(89.4) |
|  | Missing | 206(0.3) | 105(0.4) | 220(0.5) | 208(0.5) |
| Health care tasks |  |  |  |  |  |
|  | No | 21770(36.7) | 9791(35) | 16634(36.5) | 17176(37.4) |
|  | Yes | 37390(63) | 18118(64.7) | 28767(63.1) | 28539(62.1) |
|  | Missing | 206(0.3) | 105(0.4) | 220(0.5) | 208(0.5) |
| Home maintenance |  |  |  |  |  |
|  | No | 19700(33.2) | 8924(31.9) | 14630(32.1) | 13904(30.3) |
|  | Yes | 39460(66.5) | 18985(67.8) | 30771(67.4) | 31811(69.3) |
|  | Missing | 206(0.3) | 105(0.4) | 220(0.5) | 208(0.5) |
| Meals |  |  |  |  |  |
|  | No | 13975(23.5) | 6543(23.4) | 11454(25.1) | 11704(25.5) |
|  | Yes | 45185(76.1) | 21366(76.3) | 33947(74.4) | 34011(74.1) |
|  | Missing | 206(0.3) | 105(0.4) | 220(0.5) | 208(0.5) |
| Movement activities |  |  |  |  |  |
|  | No | 51374(86.5) | 24102(86) | 38844(85.1) | 38514(83.9) |
|  | Yes | 7786(13.1) | 3807(13.6) | 6557(14.4) | 7201(15.7) |
|  | Missing | 206(0.3) | 105(0.4) | 220(0.5) | 208(0.5) |
| Self-care |  |  |  |  |  |
|  | No | 31958(53.8) | 15239(54.4) | 25500(55.9) | 25631(55.8) |
|  | Yes | 27202(45.8) | 12670(45.2) | 19901(43.6) | 20084(43.7) |
|  | Missing | 206(0.3) | 105(0.4) | 220(0.5) | 208(0.5) |
| Social and community participation |  |  |  |  |  |
|  | No | 15539(26.2) | 7072(25.2) | 12411(27.2) | 12433(27.1) |
|  | Yes | 43621(73.5) | 20837(74.4) | 32990(72.3) | 33282(72.5) |
|  | Missing | 206(0.3) | 105(0.4) | 220(0.5) | 208(0.5) |
| Transport |  |  |  |  |  |
|  | No | 10686(18) | 4878(17.4) | 8689(19) | 8502(18.5) |
|  | Yes | 48474(81.7) | 23031(82.2) | 36712(80.5) | 37213(81) |
|  | Missing | 206(0.3) | 105(0.4) | 220(0.5) | 208(0.5) |
| Moving around places at or away from home |  |  |  |  |  |
|  | No | 33016(55.6) | 15483(55.3) | 25282(55.4) | 24884(54.2) |
|  | Yes | 26144(44) | 12426(44.4) | 20119(44.1) | 20831(45.4) |
|  | Missing | 206(0.3) | 105(0.4) | 220(0.5) | 208(0.5) |
| Not applicable |  |  |  |  |  |
|  | No | 59203(99.7) | 27938(99.7) | 45446(99.6) | 45811(99.8) |
|  | Yes | 163(0.3) | 76(0.3) | 175(0.4) | 112(0.2) |
| None |  |  |  |  |  |
|  | No | 57384(96.7) | 27105(96.8) | 43934(96.3) | 44391(96.7) |
|  | Yes | 1776(3) | 804(2.9) | 1467(3.2) | 1324(2.9) |
|  | Missing | 206(0.3) | 105(0.4) | 220(0.5) | 208(0.5) |
| Not stated |  |  |  |  |  |
|  | No | 59160(99.7) | 27909(99.6) | 45401(99.5) | 45715(99.5) |
|  | Yes | 206(0.3) | 105(0.4) | 220(0.5) | 208(0.5) |
| Other |  |  |  |  |  |
|  | No | 56127(94.5) | 26189(93.5) | 41625(91.2) | 41580(90.5) |
|  | Yes | 3033(5.1) | 1720(6.1) | 3776(8.3) | 4135(9) |
|  | Missing | 206(0.3) | 105(0.4) | 220(0.5) | 208(0.5) |

IQR=Inter quartile range; SD= Standard deviation

**Australian States**

ACT=Australian Capital Territory; NSW= New South Wales; NT=Northern Territory; QLD=Queensland; SA=South Australia; TAS= Tasmania; VIC=Victoria; WA=Western Australia

**Home care packages (2003-2013)**

CACP=Community Aged Care Package; EACH=Extended Aged Care at Home; EACHD=Extended Aged Care at Home Dementia.

**Home care packages (Since 2014)**

Level1 = Home Care Level 1; Level2 = Home Care Level 2; Level3 = Home Care Level 3; Level4 = Home Care Level 4.

**English proficiency (EP) group classification**

EP0 = Australian born; EP1 = Born in countries rating 98.5% or higher on the EP index with at least 10,000 residents in Australia; EP2 = Born in countries rating 84.5% or higher on the EP index, other than those in EP1; EP3 = Born in countries rating 57.5% to less than 84.5% on the EP index; EP4 = Born in countries rating less than 57.5% on the EP index.
