# Supplementary material for: Prolonged Wait Time Prior to Entry to Home Care Packages Increases the Risk of Mortality and Transition to Permanent Residential Aged Care Services: Findings from the Registry of Older South Australians (ROSA)
Source: J Nutr Health Aging. 2018 Dec 4;23(3):271–80. doi: 10.1007/s12603-018-1145-y (PMC6399871; doi:10.1007/s12603-018-1145-y)
Supplement: Supplementary file 2 — Supplementary material, approximately 41.9 KB. [file mmc2.docx]

**Supplementary Table 2.** Cumulative survival probability after accessing a home care package by wait time

|  |  |  |  | Cumulative survival probability (95%CI) at each time period | | | | | | |
| --- | --- | --- | --- | --- | --- | --- | --- | --- | --- | --- |
| Wait time | **N** | **Observed deaths (% crude mortality)** | **Median follow up time (years) for the entire period [IQR]** | | **30 days, N=177081** | **90 days, N=172090** | **6 months, N=165162** | **1-year, N=152265** | **2 years,**  **N=123133** | **5 years, N=42119** |
| 0-30 days | 59366 | 38157(64.3) | 4.0(1.7, 7.4) | | 0.99(0.98, 0.99) | 0.95(0.95, 0.95) | 0.91(0.91, 0.91) | 0.84(0.84, 0.84) | 0.72(0.72, 0.72) | 0.41(0.41, 0.42) |
| 31-59 days | 28014 | 17976(64.2) | 3.9(1.8, 7.2) | | 0.99(0.99, 0.99) | 0.96(0.96, 0.96) | 0.92(0.92, 0.92) | 0.85(0.84, 0.85) | 0.72(0.72, 0.73) | 0.40(0.39, 0.41) |
| 2-6 months | 45621 | 28427(62.3) | 4.1(1.9, 7.3) | | 0.99(0.99, 0.99) | 0.97(0.97, 0.97) | 0.93(0.93, 0.94) | 0.86(0.86, 0.87) | 0.73(0.73, 0.74) | 0.41(0.41, 0.42) |
| Over 6 months | 45923 | 26928(58.6) | 3.8(1.7, 6.9) | | 0.99(0.99, 0.99) | 0.97(0.97, 0.97) | 0.93(0.92, 0.93) | 0.85(0.85, 0.86) | 0.71(0.71, 0.72) | 0.38(0.38, 0.39) |
| All | 178924 | 111488(62.3) | 4.0(1.8, 7.2) | | 0.99(0.99, 0.99) | 0.96(0.96, 0.96) | 0.92(0.92, 0.92) | 0.85(0.85, 0.85) | 0.72(0.72, 0.73) | 0.40(0.40, 0.41) |

**Supplementary Table 3:** Cumulative incidence of transition to permanent residential aged care after a home care package by wait time

| Wait time | N | N (%) transitioned into permanent residential care | Median time (years) between home and permanent residential care [IQR] | Cumulative incidence (95%CI) of transition at each time-period | | | | | |
| --- | --- | --- | --- | --- | --- | --- | --- | --- | --- |
|  |  |  |  | **30 days,**  **N=91086** | **3 months,**  **N=82787** | **6 months,**  **N=69891** | **1 year,**  **N=50055** | **2 year,**  **N=26398** | **5 year,**  **N=4186** |
| 0-30 days | 59366 | 31127(52.4) | 2.9(1.0, 9.0) | 0.01(0.01, 0.01) | 0.06(0.06, 0.06) | 0.14(0.14, 0.14) | 0.26(0.25, 0.26) | 0.42(0.41, 0.42) | 0.64(0.63, 0.64) |
| 31-59 days | 28014 | 14949(53.4) | 2.8(1.0, 7.7) | 0.01(0.01, 0.01) | 0.06(0.05, 0.06) | 0.13(0.13, 0.14) | 0.26(0.25, 0.26) | 0.42(0.42, 0.43) | 0.65(0.64, 0.66) |
| 2-6 months | 45621 | 24224(53.1) | 2.8(1.0, 7.7) | 0.01(0.01, 0.01) | 0.05(0.05, 0.06) | 0.13(0.12, 0.13) | 0.25(0.25, 0.26) | 0.41(0.41, 0.42) | 0.65(0.64, 0.65) |
| Over 6 months | 45923 | 22687(49.4) | 2.8(1.0, 7.5) | 0.01(0.01, 0.01) | 0.06(0.06, 0.06) | 0.14(0.14, 0.14) | 0.26(0.25, 0.26) | 0.42(0.41, 0.42) | 0.66(0.65, 0.66) |
| All | 178924 | 92987(52.0) | 2.8(1.0, 8.0) | 0.01(0.01, 0.01) | 0.06(0.06, 0.06) | 0.14(0.13, 0.14) | 0.26(0.26, 0.26) | 0.42(0.41, 0.42) | 0.65(0.64, 0.65) |
