# Supplementary material for: Prolonged Wait Time Prior to Entry to Home Care Packages Increases the Risk of Mortality and Transition to Permanent Residential Aged Care Services: Findings from the Registry of Older South Australians (ROSA)
Source: J Nutr Health Aging. 2018 Dec 4;23(3):271–80. doi: 10.1007/s12603-018-1145-y (PMC6399871; doi:10.1007/s12603-018-1145-y)
Supplement: Supplementary file 3 — Supplementary material, approximately 158 KB. [file mmc3.docx]

**Supplementary Figure 1a:** Proportion of individuals approved for home care packages stratified by type of package received at entry to services

**
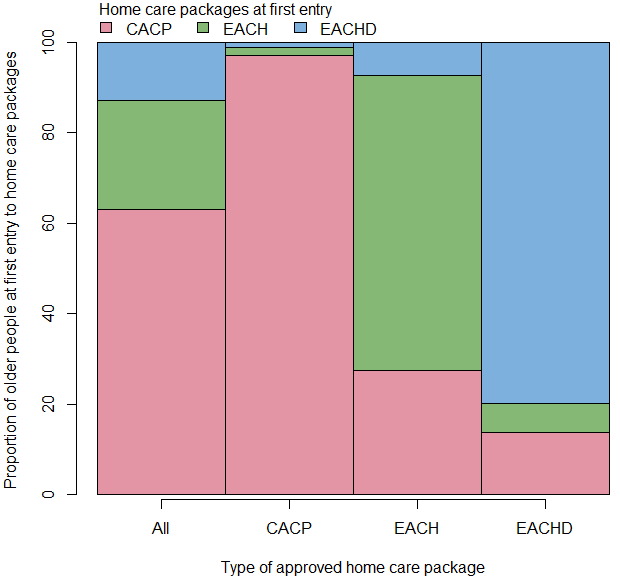
**

**Supplementary Figure 1b:** Proportion of older people who had approval for home care packages by their wait time

**
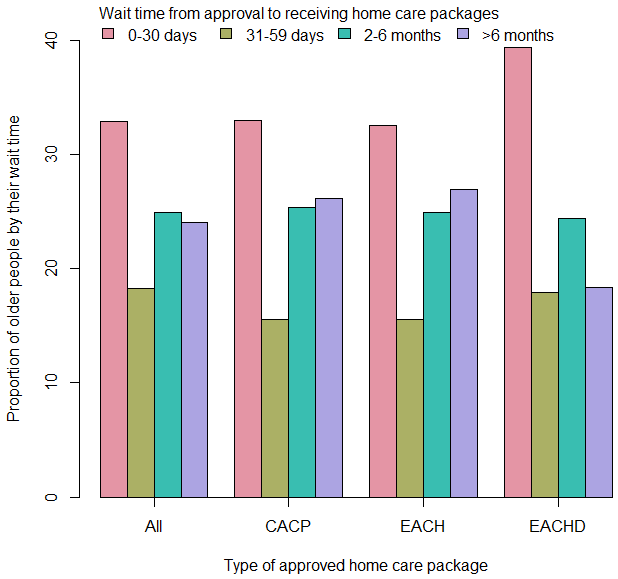
**

**Supplementary Figure 1c:** Proportion older people entering home care packages stratified by their wait time

**
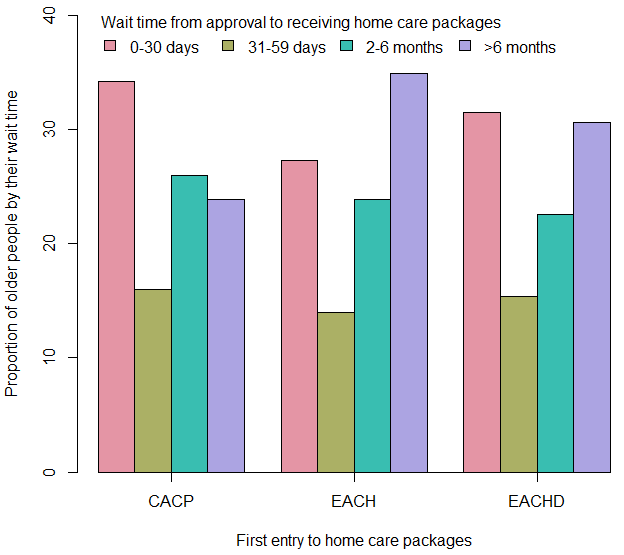
**

**Supplementary Figure 1d:** Distribution of wait times by state


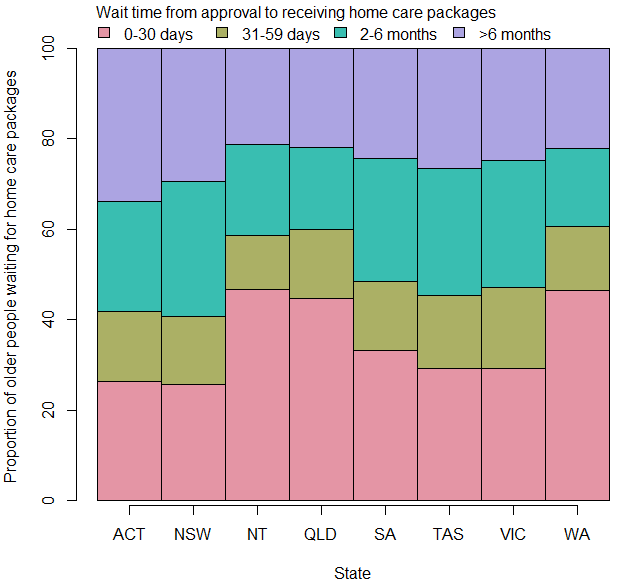


**Supplementary Figure 1e:** Proportion of people assessed for a home care pakcage service stratified by their wait time and by year **
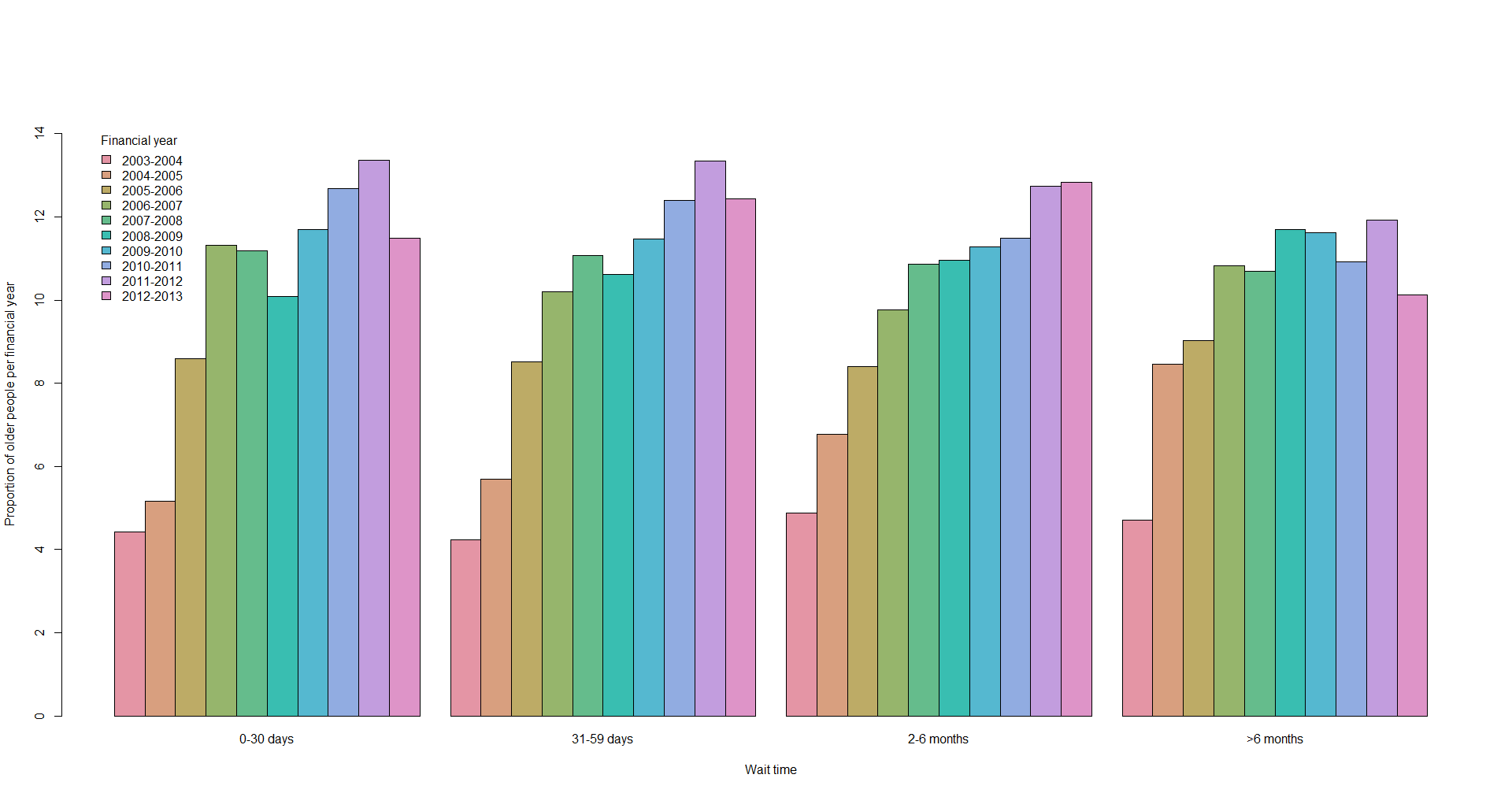
**
